# Genome data for 25 isolates of the prevalent forest and nursery pathogen, *Phytophthora cinnamomi*, from New Zealand

**DOI:** 10.1016/j.dib.2025.111655

**Published:** 2025-05-15

**Authors:** Rebecca L. McDougal, Kerehi Ruaine, Gayathri Vaidyanathan, Jason Shiller, Tancred Frickey, Darryl Herron, Rosie Bradshaw

**Affiliations:** a Scion (The New Zealand Forest Research Institute Ltd.), Titokorangi Drive, Private Bag 3020, Rotorua 3046, New Zealand; b BioProtection Aotearoa, New Zealand; c The New Zealand Institute for Plant and Food Research Ltd, Te Puke, New Zealand; d School of Food Technology and Natural Sciences, Massey University, Palmerston North 4410, New Zealand

**Keywords:** Forest disease, Forest pathogen, Nursery pathogen, Genome sequence, *Phytophthora*

## Abstract

*Phytophthora cinnamomi* is a globally distributed plant pathogen with a remarkably broad host range. It is considered one of the greatest threats to natural and productive ecosystems, especially under changing climatic conditions. In New Zealand, this pathogen has been present for a long time, but the genetic diversity is not well understood. We describe here draft genome assemblies for 25 isolates of *P. cinnamomi* from New Zealand trees, forests and nurseries. The genome assembly data showed an average size of 129 MB with an average N90 value of 297,992 bp. This data will enable the analysis of genetic diversity of New Zealand *P. cinnamomi* isolates, and the development of accurate detection and diagnostic tools.

Specifications Table

**Table:** 

Subject	Biology
Specific subject area	Oomycete plant pathogen genomics
Type of data	Genomic data, presented in Tables
Data collection	Genomics data for twenty-five *Phytophthora cinnamomi* isolates from various plant hosts in New Zealand were generated from purified and sequenced DNA. The genomic DNA was sequenced on an Illumina NovaSeq 6000 sequencing platform.
Data source location	Scion (The New Zealand Forest Research Institute Ltd.), Titokorangi Drive, Private Bag 3020, Rotorua 3046, New Zealand
Data accessibility	Repository name: NCBI Data identification number: BioProject number PRJNA1197507. Direct URL to data: https://www.ncbi.nlm.nih.gov/bioproject/PRJNA1197507/ The data has been deposited in GenBank SRA archive found at NCBI, meeting their requirements for submission.
Related research article	Not applicable.

## 1 Value of the Data

• This report describes genomic data from a collection of 25 *Phytophthora cinnamomi* isolates from various native and exotic trees and woody shrubs in New Zealand, as well as avocado.
• *Phytophthora cinnamomi* is a ubiquitous forest, horticulture and nursery pathogen in New Zealand with a long association with forestry.
• Globally *P. cinnamomi* has a very wide distribution and host range and is responsible for devastating forest and horticultural diseases.
• The genomic data provided in this study will facilitate future studies to address the genetic population diversity, evolutionary analyses and host-pathogen interactions*.*
• Predicting the evolution of pathogens under changing climatic conditions is critical to mitigate future risk.

## 2 Background

*Phytophthora cinnamomi* Rands, first isolated in 1922 from cinnamon trees in Sumatra, now has a worldwide distribution, infecting more than 3000 plant hosts [1]. In New Zealand, *P. cinnamomi* has been long associated with plants and trees in many land use systems [2], including exotic and native trees, forests and plant nurseries [[3], [4], [5]]. *Phytophthora cinnamomi* has caused devastating plant and tree losses including local extinctions, flow-on biodiversity impacts and ecosystem collapse in some cases, with limited options for eradication [1,[6], [7], [8]]. It is considered one of the top 100 worst alien invasive species [9] and this ‘biological bulldozer’ [10] is ranked number seven of the top 10 oomycete pathogens in plant pathology [11].

Genomic resources for *P. cinnamomi* are available from a number of different hosts from across the globe [12,13]. A larger resource pool, including broad hosts from New Zealand, enables analysis of the genetic diversity, evolutionary potential and the risk this pathogen may pose under conditions of climate change [14,15]. The isolates used in this study were collected as part of past and present biosecurity surveillance and forest research programs in New Zealand and are housed and maintained in Scion’s National Forest Culture Collection (NZFS). Isolates were selected to capture differences in temporal, geographic or host association.

## 3 Data Description

This data article is comprised of genomics resources of a large collection of *Phytophthora cinnamomi* isolates (Table 1) [16], with draft genome assemblies (Table 2). The average size of the genomes was 129 MB with an average N90 value of 297,992 bp. The quality of the genome assemblies, using BUSCO (Benchmarking Universal Single-Copy Orthologs) and the Stramenopile database [17], demonstrated 98–100 % completeness (Table 2). The assembly data presented here provides a resource for future population and genomic analyses, as well as the development of diagnostic tools.

**Table 1:** *Phytophthora cinnamomi* isolates used in this study.

NZFS^a^	Host	Substrate	Year Collected	Region of New Zealand	BioSample Accession
102	*Larix decidua*	soil	1968	Nelson	SAMN45808063
102.16	*Pinus radiata*	soil	1999	Northland	SAMN45808062
609	*Eucalyptus nitens*^b^	stem tissue	2001	Taupo	SAMN45808064
627	*Pinus lambertiana*^b^	roots	2001	Bay of Plenty	SAMN45808057
939	*Betula pendula*	soil	2003	Auckland	SAMN45808044
979	*Quercus robur*	soil	2003	Auckland	SAMN45808049
1012	*Quercus palustris*	stem lesion	2003	Auckland	SAMN45808048
2316	*Pinus radiata*	soil	2005	Auckland	SAMN45808046
2545	*Xeronema callistemon*	soil and roots	2005	Auckland	SAMN45808050
2657	*Pseudotsuga menziesii*^b^	plant tissues and soil	2006	Taupo	SAMN45808066
2960	*Araucaria heterophylla*	soil	2007	Auckland	SAMN45808043
3030	*Agathis australis*	soil	2008	Auckland	SAMN45808042
3876	*Agathis australis*	soil and roots	2014	Bay of Plenty	SAMN45808051
4342	*Persea americana*	soil	2016	Northland	SAMN45808060
4359	*Persea americana*	soil	2016	Northland	SAMN45808061
4411	*Persea americana*	soil	2017	Bay of Plenty	SAMN45808054
4424	*Persea americana*	soil	2017	Bay of Plenty	SAMN45808055
4458	*Persea americana*	soil	2017	Bay of Plenty	SAMN45808056
4801	*Gevuina avellana*^b^	root collar	2019	Bay of Plenty	SAMN45808052
4805	*Coprosma robusta*	soil and roots	2019	Auckland	SAMN45808045
5108	*Quercus* sp.	stem canker	2020	Bay of Plenty	SAMN45808058
5347	*Pinus radiata*	soil	2021	Auckland	SAMN45808047
5425	*Knightia excelsa*^b^	soil	2021	Bay of Plenty	SAMN45808053
5434	*Pinus radiata*	root collar	2022	Taupo	SAMN45808065
5436	*Vitex lucens*	soil	2021	Bay of Plenty	SAMN45808059

a National Forest Culture Collection, Scion, Rotorua, New Zealand (NZFS)

b Isolated from a plant production nursery

**Table 2:** Summarised genome information for 25 *Phytophthora cinnamomi* isolates.

Assembly Statistics	*Phytophthora cinnamomi* NZFS isolate number												
	102	102.16	609	627	939	979	1012	2316	2545	2657	2960	3030	3876
Number of scaffolds > 50 Kb	118	119	119	117	120	121	121	123	123	121	125	121	117
Assembly length (Mb)	142.41	105.99	146.28	143.10	140.67	144.05	124.24	122.10	121.39	145.00	152.01	126.36	123.12
Largest Scaffold size (Kb)	2934	2962	3085	2927	3068	3093	2994	2994	3021	3094	3094	3110	2888
N50 length (bp)	324700	279998	2624	4089	6816	4838	231864	242485	246648	4081	5725	230416	41359
N90 length (bp)	431292	162592	426594	439703	393586	410383	251731	239442	237589	414543	418820	257952	260417
GC%	54.28	53.93	54.31	54.21	54.41	54.04	54.02	54.11	53.79	54.19	53.91	53.83	53.78
Complete & single copy BUSCOs (%)^a^	100	99	100	98	100	100	100	99	99	99	99	99	98
Complete & duplicated BUSCOs (%)	0	0	0	0	0	0	0	0	0	0	0	1	1
Fragmented BUSCOs (%)	0	0	0	2	0	0	0	1	1	1	1	0	1
Missing BUSCOs (%)	0	1	0	0	0	0	0	0	0	0	0	0	0

Assembly Statistics (cont’d)	4342	4359	4411	4424	4458	4801	4805	5108	5425	5434	5436	5437	

Number of scaffolds > 50 Kb	119	122	122	121	122	121	122	122	121	117	121	121	
Assembly length (Mb)	122.92	112.33	125.32	154.80	121.75	127.80	126.09	110.56	126.26	124.03	112.56	124.03	
Largest Scaffold size (Kb)	2908	2979	3079	3077	3126	3007	3127	3014	3131	2934	2993	3059	
N50 length (bp)	55152	298090	233226	382300	251607	224449	226231	290738	230610	34369	296951	238255	
N90 length (bp)	259132	189187	254016	495686	237515	275360	257685	184380	257123	263961	183046	248088	
GC%	53.9	53.72	53.79	54.65	53.76	53.91	53.88	53.95	53.81	53.81	53.79	53.77	
Complete & single copy BUSCOs (%)	100	100	99	99	99	98	99	100	99	100	99	99	
Complete & duplicated BUSCOs (%)	0	0	0	0	0	1	0	0	0	0	0	0	
Fragmented BUSCOs (%)	0	0	1	1	1	1	1	0	1	0	1	1	
Missing BUSCOs (%)	0	0	0	0	0	0	0	0	0	0	0	0	

a Benchmarking universal single-copy orthologs (BUSCO) [17]

## 4 Experimental Design, Materials and Methods

### 4.1 Isolate preparation, growth and DNA extraction

Cultures for 25 *P. cinnamomi* isolates (Table 1) were revived from the NZFS collection and hyphal tipping was performed to ensure cultures were pure [18]. The identity of the cultures was confirmed using end-point PCR with *P. cinnamomi* species-specific primers Ycin3F and Ycin4R [19].

Cultures were grown on 80–100 ml clarified carrot medium for 14–21 days at 20 °C. Cultures were washed three times in sterile distilled water and frozen overnight at −80 °C prior to freeze-drying mycelium for 24–48 h. Tissue lysis was performed by grinding mycelium with liquid nitrogen in sterile mortar and pestles and DNA was extracted with a Qiagen DNeasy Plant Maxi Kit, eluting in 2 ml nuclease-free water. DNA was purified and concentrated using AMPure XP beads according to the manufacturer’s instructions (Beckman Coulter Life Sciences, Australia). DNA was quantified using a Qubit dsDNA BR Assay where concentrations ranged from 31.2 to 830 ng/ul and the DNA quality was assessed using a Nanodrop where 260/280 ratios ranged from 1.42 to 1.9.

### 4.2 DNA sequencing and genome assembly

DNA libraries were prepared using NEBNext® Ultra™ IIDNA Library Prep Kit (New England BioLabs, MA, USA). The genomic DNA from each isolate was sequenced using paired 150-nt Illumina reads on the Novaseq™ 6000 platform at Novogene Co., Ltd. (Beijing, China), via the Massey University Genome Service (Palmerston North, New Zealand). DNA sequence quality was assessed using FastQC (version 0.11.9) [20]. Genome data were assembled *de novo* using SPAdes v 3.5.0 [21] and RagTag [22]. Completeness of the genome assemblies was assessed using BUSCO (Benchmarking Universal Single-Copy Orthologs) using the Stramenopile database [17]. Raw sequence data are available via GenBank and the BioSample accession numbers listed in Table 1. The summarised genome assembly statistics are presented in Table 2.

## Limitations

Not applicable.

## Ethics Statement

This work does not involve human subjects, animal experiments, or any data collected from social media platforms.

## Credit Author Statement

**RLM:** Conceptualization and design of the project, funding acquisition, methodology and data curation, project administration, supervision and writing – original draft**; KR:** methodology and formal analyses including bioinformatic analysis, **GV:** methodology and formal analysis**, JS, TF:** formal analysis, supervision, and resources, **DH:** data curation, supervision and delivery, **RB:** data curation, project administration including responsibility for overall program delivery. All authors contributed to the writing and reviewing of the manuscript.

## Data Availability

NCBIExploring the Molecular Basis of Host Specificity (Original data).
